# Investigating the Effect and Mechanism of Protocatechuic Aldehyde on Vascular Dementia Based on Multi-Omics Approach

**DOI:** 10.3390/biom16030411

**Published:** 2026-03-11

**Authors:** Tian Xiao, Jie Tao, Jiaoyang Tan, Xingjie Yi, Ziyi Wang, Xiaohua Duan

**Affiliations:** Yunnan Key Laboratory of Dai and Yi Medicines, Yunnan University of Chinese Medicine, Kunming 650500, China; xiaotian@ynucm.edu.cn (T.X.); taojie@ynucm.edu.cn (J.T.); tanjiaoyang@ynucm.edu.cn (J.T.); yixingjie@ynucm.edu.cn (X.Y.); wangziyi@ynucm.edu.cn (Z.W.)

**Keywords:** protocatechualdehyde, vascular dementia, glutamate metabolism, lactate shuttle, multi-omics

## Abstract

**Background:** The therapeutic effect and mechanism of protocatechuic aldehyde (PAL) on vascular dementia (VaD) were studied from a multi-group perspective. **Methods:** The pharmacological property of PAL was assessed by using both an in vivo two-vessel occlusion (2VO) rat model and an in vitro astrocyte–neuron co-culture system with oxygen–glucose deprivation (OGD) injury. On the basis of neurobehavioral test, Morris’ water maze test and hematoxylin and eosin staining, the pathological transformation of cognitive function and ischemic cerebral tissue was assessed. Key metabolites and targets through the comprehensive analysis of brain tissue and plasma metabolomics and transcriptomics were screened. Western blot and immunofluorescence were measured to assess proteins related to glutamate release, lactate shuttle and glycolysis. **Results:** PAL markedly improved the cognitive dysfunction of 2VO rats and reduced the nerve function score. The degeneration of neurons in the Hippocampal CA1 region was appreciably reduced. A total of eight common metabolites, including L-glutamate and L-glutamine, have been identified from plasma and brain sources. The pathway enrichment of glutamate metabolism is closely related to multiple energy metabolic pathways related to glycolysis. Combined with transcriptomic analysis and in vivo experiments, it was found that PAL can significantly downregulate the expression of the glutamate-releasing protein vGLUT1 and promote the process of glutamate transformation into glutamine. At the same time, it enhances the expression of lactate production, shuttle and utilization of related proteins GLUT-1, HK2, PFK, LDHA/B and PDH, MCT1/2/4. In the subsequent cell co-culture system, we confirmed that PAL can effectively lower the expression of vGLUT1, reduce the content of glutamate, and promote the lactate shuttle process, thus increasing the content of lactate and ATP and reducing apoptosis. **Conclusions:** PAL is associated with upregulation of key glycolytic enzymes and MCTs, suggesting a potential enhancement of the lactate shuttle mechanism. This process may involve the regulation of glutamate metabolism and coordinated modulation of energy metabolism pathways such as glycolysis, thereby improving intercellular energy supply and contributing to the therapeutic effects observed in vascular dementia. This study provides a mechanistic basis and preclinical evidence for the clinical development of PAL.

## 1. Introduction

Vascular dementia (VaD) is the second most common subtype of dementia after Alzheimer’s disease, which mainly affects the elderly [1]. With the acceleration of the global population aging trend, the World Health Organization predicts that the number of dementia patients worldwide will triple by 2050, and the incidence and disability rate of VaD will also increase significantly [2]. VaD refers to the syndrome of cognitive impairment caused by cerebrovascular disease. Patients with VaD are usually characterized by executive dysfunction, which leads to a gradual decline in daily life ability and gradually erodes their independence and dignity [3]. At present, the clinical management of VaD mainly focuses on controlling vascular risk factors and alleviating cognitive symptoms [4]. Although these methods can slow down the progression of the disease or partially relieve symptoms, they do not fundamentally solve the problems of neuronal damage and cognitive decline [5].

Monomer extracts from traditional medicines have significant advantages in the treatment of VaD, including significant efficacy and high safety, making them suitable for long-term use [6]. *Gastrodia elata* is a traditional Chinese medicine. Its active ingredient protocatechuic aldehyde (C_7_H_6_O_3_, PAL) can significantly improve cognitive defects and enhance learning and memory [7]. Unlike single-target drugs, PAL protects against cerebral ischemia injury through a multifaceted mechanism involving the regulation of multiple signaling pathways. PAL activates the PKCε/Nrf2/HO-1 pathway to combat oxidative stress, as its neuroprotection is abolished by PKCε or Nrf2 knockdown. Concurrently, PAL triggers PINK1 Parkin-mediated mitophagy via the OGT-PINK1 pathway, promoting mitochondrial network health by enhancing Opa1 and inhibiting Drp1. PAL also modulates the HIF1α PDK1 axis to restore energy metabolism, reversing CI/R-induced metabolic shifts and increasing ATP production. Regarding cell death, PAL inhibits NLRP3-mediated pyroptosis, a process involving GSDMD, Caspase-1, and IL-1β, in a manner dependent on lncRNA Xist. Additionally, PAL preserves BBB integrity by upregulating tight junction proteins, including occludin and claudin-5, and downregulating AQP-4 and MMP-3, while inhibiting NO synthase activity. These integrated pathways collectively reduce infarct volume, improve neurological outcomes, and promote cell survival [8,9]. Simultaneously, studies indicate that the brain uptake of PAL is enhanced under pathological conditions of cerebral ischemia [10]. These properties make it a promising candidate for development as a neuroprotective agent to improve cognitive function. Although PAL has been clearly demonstrated to exert beneficial effects in the context of cerebral ischemia, the specific pathways through which it alleviates neural injury, particularly in models of chronic hypoperfusion, remain unreported. This study aims not only to explore whether PAL can ameliorate cognitive dysfunction, but also, more importantly, to investigate its potential underlying mechanisms of action.

Multi-omics research is a multi-level, multi-dimensional analytical method that integrates various types of omics data to comprehensively reveal the molecular mechanisms and regulatory networks of multi-target drugs in the intervention of diseases [11]. The plasma metabolomics reflects the systemic metabolic state of the animals, while brain tissue metabolomics directly reveals metabolic changes in the brain. Through an integrated analysis, the common metabolites in the plasma and brain tissue and their changing patterns can be identified [12]. Further combining transcriptomics data allows the revelation of molecular disease mechanisms from both gene expression and metabolic regulation perspectives, providing new insights and methods for PAL treatment [13].

Therefore, this study utilized a 2VO rat model and an oxygen–glucose deprivation (OGD) injury model using an astrocyte–neuron co-culture system, employing plasma and brain tissue metabolomics combined with a transcriptomics analysis to unravel the therapeutic properties of PAL in treating VaD and decipher its mechanisms of neuroprotection.

## 2. Materials and Methods

### 2.1. Experimental Animals

Male Sprague–Dawley rats, weighing 250–280 g, 8 weeks old, were purchased from Sibeifu (Beijing) Biotechnology Co., Ltd. (Beijing, China), License number SCXK (Jing) 2024-0001. Pregnant female Sprague–Dawley rats, gestational day 10–12, were provided by Jiangsu Huachuang Xinnuo Medical Technology Co., Ltd. (Taizhou, China), License number: SYXK (Su) 2020-0009. All animals were maintained in an SPF environment at 22 ± 2 °C with ad libitum access to food and water, and all procedures were conducted in accordance with guidelines approved by the Animal Ethics Committee of Yunnan University of Chinese Medicine (Approval number: YNUCM-XMSB-G-20250023).

### 2.2. Animal Grouping, Dosing, and 2VO Model Replication

Notably, 40 rats were evenly assigned into four groups: sham, model, PAL high-dose, and low-dose. Out of the 10 rats in each group, 4 were used for histological staining analysis, 3 were used for fresh brain tissue sampling for metabolomics and transcriptomics, and 6 were used for fresh brain tissue sampling for biochemical and Western blot analyses. We reported that the drug doses for the PAL high- and low-dose groups were set at 20 mg/kg and 10 mg/kg (AB1582, Chengdu Alfa Biotechnology Co., Ltd., Chengdu, China), respectively, based on preliminary pilot results and the effective dosage range of PAL previously reported in other cerebral ischemia models. Since the 20 mg/kg dose demonstrated favorable outcomes in phenotypic indicators, it was selected as the primary dose for subsequent in vivo mechanistic investigations. The sham and model received an equal volume of distilled water. Intragastric administration was performed once daily for 28 days after the commencement of modeling.

Rats in all groups underwent 2VO model replication, except for rats in the sham group. The rats were following anesthesia induction with 5% isoflurane (20260311, RWD Life Science) for induction and maintained with 1% isoflurane. The neck was incised along the midline, and the surgically exposed bilateral common carotid arteries and double-ligated proximally and distally. The wound was sutured and disinfected with erythromycin ointment.

### 2.3. Neurological Function Scoring and Water Maze Test

Neurological behavior was scored in modeled rats using the Zea Longa 5-point scale. The 0 points indicated no neurological deficits, with both forelimbs extending fully towards the floor; 1 point was defined as an inability to fully extend the contralateral forelimb; 2 points were assigned for circling movement; 3 points indicated leaning to one side; 4 points were assigned for an inability to walk spontaneously, indicating the loss of motor ability.

After the drug was given, a 4-day positioning navigation test was performed. On days 1–4, put the rats into water from different quadrants, and the test time was 90 s. The escape latency is measured as the time required to find the hidden platform. The rat is positioned on the platform for 15 s to learn its location, and a 90 s latency is assigned, if the platform is not located during the 90 s trial. The average escape incubation period of 4 days was recorded as the escape latency of the navigation test. After the navigation test, the hidden platform was withdrawn on the 5th day for the space exploration test. Facing the wall of the first quadrant, each rat was placed in the water for the 120 s test duration. Record the number of times the rats passed through the original platform position and the total swimming distance and measure the cognitive function by evaluating the spatial memory and learning ability.

### 2.4. Hematoxylin and Eosin Staining

The prepared brain tissue paraffin sections were deparaffinized using xylene. Rehydration was performed using a graded series of ethanol from 100% to 75% and distilled water. The nuclei were processed with hematoxylin solution (G1076, Wuhan Servicebio Technology Co., Ltd., Wuhan, China) for 3 min and then with 1% hydrochloric acid alcohol for 30 s for differentiation. The cytoplasm was stained with 1% eosin solution (G1076, Wuhan Servicebio Technology Co., Ltd) for 1 min. Dehydration was carried out using 80–100% ethanol, and sections were cleared by adding xylene. Observation under an optical microscope (SWE-CX63, Wuhan Servicebio Technology Co., Ltd.) constituted the final step, performed on sections that had been mounted with neutral balsam. Neuronal survival density in the CA1 region was determined by counting the number of surviving neurons. Only cells with an intact morphology and a clearly visible nucleolus were included in the counts. For each tissue section, three non-overlapping fields of view were randomly selected, photographed, and quantified. The average value from these three fields was used as the representative value for that section.

### 2.5. Detection of Brain Tissue Physiological and Biochemical Indicators

Hippocampal brain tissue was obtained from rats, and all steps were conducted in strict accordance with the protocols specified by the biochemical and ELISA kits. The contents of Ca^2+^, glutamine (BTK189, BTK133, Bioswamp Life Science Lab, Wuhan, China), lactate, glutamate (GLU-W96-N1620, Shanghai Enzyme-linked Biotechnology Co., Ltd., Shanghai, China), lactate (S0208S, Beyotime Biotechnology, Beijing, China) and adenosine triphosphate (ATP; A095-2-1, Nanjing Jiancheng Bioengineering Institute, Nanjing, China), and the activities of glutamine synthetase (GS) and glutaminase (GLS; RA20664, RA20659, Bioswamp Life Science Lab, Wuhan, China) were measured in the rat hippocampal brain tissue.

### 2.6. Plasma and Brain Tissue Metabolomics

Metabolomics analysis was provided by Metware Metabolic Biotechnology Co., Ltd. (Wuhan, China) In this study, rat plasma and brain tissue samples were first extracted. Take 50 μL of plasma sample and mix it with 20% acetonitrile–methanol solution containing internal standard; take 20 mg of brain tissue sample and add 70% methanol–water solution homogeneous with internal standard. Thereafter, all samples were centrifuged at 12,000 r/min and 4 °C for 10 min. The obtained supernatant was then centrifuged again under the same conditions for 3 min and finally 200 μL of clarified supernatant was taken for analysis.

Liquid chromatography separation is carried out in the positive ion mode adopts Waters ACQUITY UPLC HSS T3 C18 column (100 mm × 2.1 mm, 1.8 μm) to contain water containing 0.1% formic acid (A) and acetonitrile containing 0.1% formic acid (B) A column temperature of 40 °C, a flow rate of 0.4 mL/min, and a 10 min gradient elution were used for the mobile phase. The negative ion mode adopts Waters ACQUITY UPLC BEH HILIC column (150 mm × 1 mm, 1.7 μm). The chromatographic conditions comprised a mobile phase of 20 mM ammonium formate solution at a specific ratio, a column temperature maintained at 40 °C, a flow rate set at 0.4 mL/min, and a gradient elution carried out over 6.51 min. The mass spectrometry uses the ESI-QTRAP-MS/MS system. The ion source temperature is 500 °C, and the air curtain air pressure is 25.0 psi. The ion spray voltage is set to +5500 V and −4500 V respectively. All data is collected in multiple reaction monitoring (MRM) mode, and polypropylene is used before analysis.

In data processing, principal component analysis is carried out first, and then differential metabolites are screened according to the standard of variable importance in projection (VIP) value > 1.0 and *t*-test *p* value < 0.05 of the orthogonal partial least squares-discriminant analysis (OPLS-DA) model. Finally, the pathway enrichment analysis was carried out through the Kyoto Encyclopedia of Genes and Genomes (KEGG) database.

### 2.7. Transcriptomics

Transcriptomics analysis was provided by Metware Metabolic Biotechnology Co., Ltd. Total RNA was extracted from brain tissue using the Trizol reagent method, with its quality and concentration subsequently assessed. Libraries that passed quality control were pooled according to their effective concentrations and the target sequencing data volume prior to sequencing. Sequencing was performed on an Illumina platform to generate paired-end 150 bp reads. High-quality clean reads obtained after quality control and filtering with fastp were aligned and mapped using the HISAT2 aligner with a pre-built index, followed by gene expression quantification via featureCounts. Based on differential expression analysis performed by DESeq2 with Benjamini–Hochberg adjusted *p*-values, DEGs were defined by an adjusted *p*-value < 0.05 and |log_2_FoldChange| > 1, followed by KEGG pathway enrichment analysis using the hypergeometric distribution test.

### 2.8. Multi-Omics Integrated Analysis

This experiment screened for common metabolites derived from the plasma and brain. Metabolites with strong clinical diagnostic significance were screened using ROC curves with an area under the curve (AUC) > 0.9 as the threshold. The SMPDB The Enrichment Analysis was conducted with the MetaboAnalyst platform (https://www.metaboanalyst.ca/, accessed on 13 July 2024).

Simultaneously, the above metabolites were imported into the Swiss Target Prediction and SymMap databases for target prediction. The Dis GENET, TTD, and OMIM databases were used to screen targets related to “VaD,” and duplicate targets were removed. Cytoscape v3.10.0 was utilized to identify the core targets associated with metabolites, based on a protein–protein interaction network that was constructed in the STRING database from the intersection of metabolite targets and disease targets. Subsequently, these core targets were intersected with the transcriptomics of differentially expressed genes to screen out the key genes involved in PAL treatment of VaD.

### 2.9. Primary Brain Cell Extraction and Cell Co-Culture

Hippocampal tissues were collected from E18 SD rat embryos under sterile conditions and dissected. The tissues were placed in HBSS (H1025, Beijing Solarbio Science & Technology Co., Ltd., Beijing, China) containing 2 mg/mL papain and 50 μg/mL DNase I (D8071, Solarbio), and mechanically dissociated at 37 °C for 20 min. Following digestion, the tissue fragments were rinsed three times with sterile HBSS to remove the digestive enzymes. The washed tissue fragments were transferred into Neurobasal-A growth medium (10888022, Gibco, Grand Island, NY, USA) supplemented with 5% fetal bovine serum (SH30070.03, Hyclone, Logan, UT, USA), 0.5 mM L-glutamine, 0.5 mM GlutaMax, 0.01% penicillin-streptomycin, and 0.02% SM1 neuronal supplement. The tissues were gently triturated using a 5 mL pipette, and the resulting cell suspension was filtered through an 80 μm mesh to obtain a single-cell suspension.

The cells were then seeded in Neurobasal-A growth medium without FBS, containing L-glutamine, GlutaMax, penicillin-streptomycin, and SM1. At 4 to 6 h post-seeding, the medium was completely replaced with fresh serum-free medium as described above to remove residual serum. Thereafter, half of the medium was replaced every two days to maintain the culture.

On day 4 post-seeding, 1.5 mM L-leucine methyl ester (HY-W037451, MedChemExpress, Shanghai, China) was added to the culture medium to deplete microglia from the astrocyte–neuron co-culture. The cells were cultured for 2 weeks before being used for subsequent experiments. Co-cultured cells were identified via immunofluorescence for NeuN/GFAP/DAPI according to Section 2.12, and positive cell counts were calculated.

### 2.10. Cell Grouping, Dosing, and OGD Model Replication

The cell experiment consisted of three groups: the control group, OGD group, and PAL group. The PAL drug concentration was 4 μg/mL, which was added 24 h before modeling and maintained until the end of the reoxygenation and reglucosis phase.

OGD model replication: In the OGD group, the normal medium was substituted for glucose-free DMEM medium at an equal volume; for the PAL group, it was substituted with an equal volume of glucose-free DMEM containing 4 μg/mL PAL; the control group was substituted with an equal volume of normal medium. Except for the control group, cells from other groups were put into a tri-gas incubator (1% O_2_, 94% N_2_, 5% CO_2_) for 6 h to induce OGD injury. The treated cells and their media were used for subsequent detection.

### 2.11. Detection of Physiological and Biochemical Indicators in Co-Cultured Cells

The media from co-cultured cells in each group were collected. All operations were conducted in strict compliance with the guidelines outlined in the biochemical kits’ instructions. The intracellular contents of glutamate (A074-1-1, Nanjing Jiancheng Bioengineering Institute), lactate, and ATP (BC2235, BC5475, Solarbio) were measured.

### 2.12. Immunofluorescence

Co-cultured cells from each group were fixed by covering the slides with 4% paraformaldehyde for 15 min. The permeabilization solution was added and incubated at 25 °C for 15 min after PBS washing. The 300 μL blocking solution was added and blocked at 25 °C for 1 h. The slides were placed in primary antibodies, including monocarboxylate Transporter 1 [MCT1], MCT2 (PA5-72957, PA5-76603, Thermo Fisher Scientific, Pittsburgh, PA, USA), MCT4, GFAP, NeuN (22787-1-AP, 60190-1-Ig, 26975-1-AP, Proteintech, Chicago, IL, USA), DAPI (4083, Cell Signaling Technology, Danvers, MA, USA), dilution ratio 1:200, and incubated at 4 °C overnight. The fluorescent secondary antibodies (Goat Anti-Mouse IgG H&L Alexa Fluor^®^ 488, Goat Anti-Rabbit IgG H&L Alexa Fluor^®^ 647, dilution ratio 1:200, ab150113, ab150079, Abcam, Cambridge, UK) were added and incubated at 25 °C for 1 h. Using a fluorescence microscope (CKX53, Olympus Corporation, Tokyo, Japan), results were documented following the addition of anti-fade mounting medium, which was applied after a light-protected PBS wash.

### 2.13. Western Blot

Notably, 100 mg of hippocampal brain tissue from each group of rats or co-cultured cells from each group were taken. Protein was extracted, quantified using a BCA kit (KGB2101, KeyGEN Bio TECH, Nanjing, China), and concentrations were adjusted before boiling for denaturation. The loading volume was 15 μL, and 5 μL of Maker (M227-01, GenStar, Beijing, China) was used. After electrophoresis, the proteins were transferred to a polyvinylidene fluoride membrane. The membrane was placed in 5% skim milk and blocked on a shaker for 1 h. The membrane was incubated with primary antibody, including vesicular glutamate Transporter 1 (vGLUT1; 1:1000, 47181, Cell Signaling Technology, Danvers, MA, USA), MCT1 (1:1000, 20139-1-AP, Proteintech, Chicago, IL, USA), MCT2 (1:500, 20355-1-AP, Proteintech, Chicago, IL, USA), MCT4 (1:2000, 22787-1-AP, Proteintech, Chicago, IL, USA) glucose transporter 1 (GLUT-1; 1:1000, 21829-1-AP, Proteintech, Chicago, IL, USA), hexokinase 2 (HK2; 1:5000, 22029-1-AP, Proteintech, Chicago, IL, USA), phosphofructokinase (PFK; 1:2000, 13389-1-AP, Proteintech, Chicago, IL, USA), pyruvate dehydrogenase (PDH; 1:1000, 2784, Cell Signaling Technology, Danvers, MA, USA), lactate dehydrogenase A (LDHA; 1:5000, 21799-1-AP, Proteintech, Chicago, IL, USA), and lactate dehydrogenase B (LDHB; 1:20,000, 14824-1-AP, Proteintech, Chicago, IL, USA) diluted in TBST at 4 °C overnight. After being washed thrice with TBST on a shaker, the membrane was incubated with secondary antibody (Goat Anti-Rabbit and Goat Anti-Mouse IgG H&L HRP; 1:10,000; ab6721, ab205719, Abcam, Cambridge, UK) diluted in TBST at room temperature for 1 h. After being washed three times with TBST on a shaker, the developer solution (32209, Thermo Fisher Scientific, Pittsburgh, PA, USA) was prepared at a 1:1 ratio, and the membrane was developed and photographed using a developer instrument (Tanon 5200, Tanon, Tokyo, Japan). Please refer to the attachment for the original WB image format.

### 2.14. Data Analysis

Statistical analyses were performed using GraphPad Prism version 9.0 (GraphPad Software, Inc., San Diego, CA, USA). Continuous data are presented as mean ± standard deviation. Prior to comparative analysis, normality of distribution was assessed. For datasets with homogeneous variance, multiple comparisons were conducted using Ordinary one-way ANOVA. In cases where variances were unequal, Brown-Forsythe and Welch ANOVA tests were employed. For data that did not follow a normal distribution, the Kruskal–Wallis test was applied. The threshold for statistical significance was set at a *p*-value of <0.05.

## 3. Results

### 3.1. Effect of PAL on Cognitive Function in 2VO Rats

In the place navigation test, the sham group exhibited a long initial escape latency. This latency gradually shortened with continued training. The model group showed a significantly longer escape latency compared to the sham group. A statistically significant difference between the groups emerged from day 2 onwards (*p* < 0.001). The model group’s latency did not show a clear decline over the following days. Administration of PAL resulted in a more pronounced decreasing trend relative to the model group. The high-dose group reached a latency similar to the sham group by day 4. The low-dose group reached a similar level by day 5. The differences between these treated groups and the sham group were not statistically significant.

**Figure 1:**
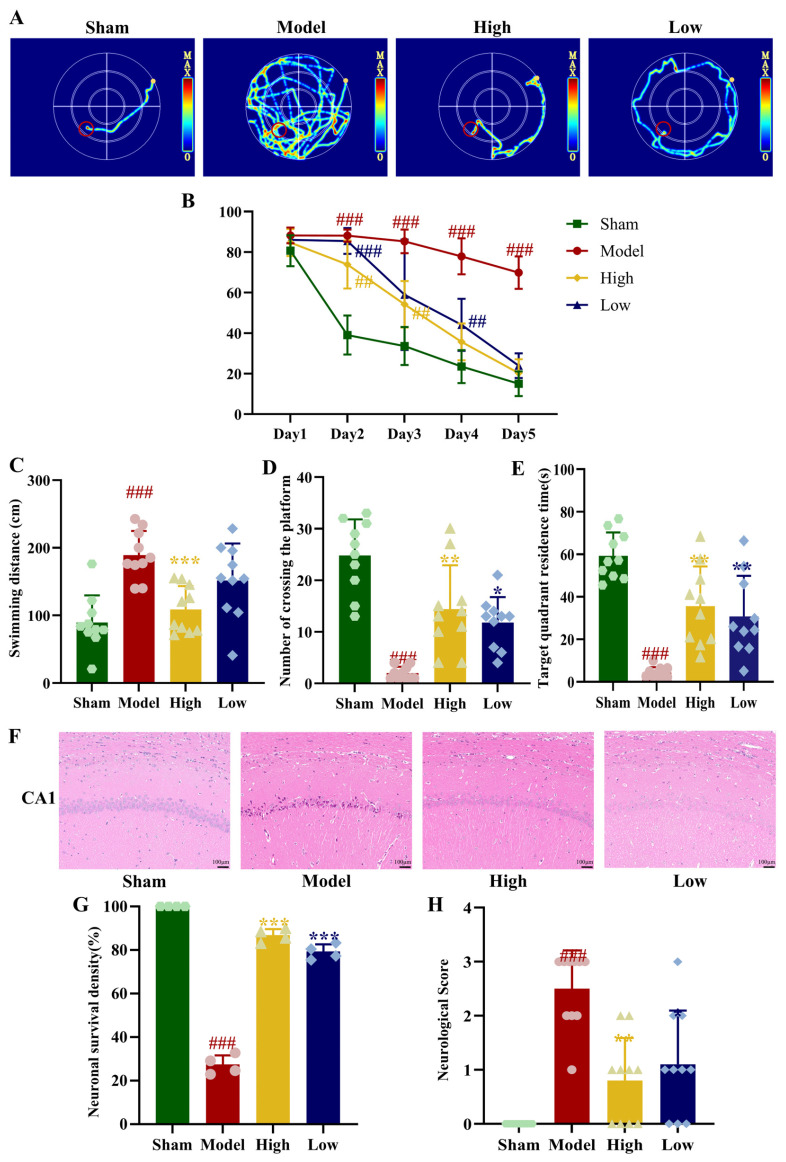
(**A**) Swimming paths of rats in the water maze for each group (*n* = 10), the red circle represents the starting point where the animals depart.; (**B**) Escape latency (2 way ANOVA, *n* = 10); (**C**) Time spent in the target quadrant (Brown-Forsythe and Welch ANOVA tests, *n* = 10); (**D**) Number of platform crossings (Kruskal–Wallis test, *n* = 10); (**E**) Swimming distance (Ordinary one-way ANOVA, *n* = 10); (**F**) Pathological images of the hippocampal CA1 region in each group (*n* = 4); (**G**) Neuronal survival density (Ordinary one-way ANOVA, *n* = 4); (**H**) Neurological scores of rats in each group (Kruskal–Wallis test, *n* = 10). In comparison to the sham group, ^###^ *p* < 0.001, ^##^ *p* < 0.01. In comparison to the model group, *** *p* < 0.001, ** *p* < 0.01, * *p* < 0.05. Data are presented as mean ± SD. High: PAL high-dose group; Low: PAL low-dose group.

In the spatial probe test, the model group rats devoted markedly less time to the target quadrant and made considerably fewer platform crossings than the sham group within 90 s. Their swimming distance was also notably longer (*p* < 0.001). Following PAL intervention, the time spent in the target quadrant and the number of platform crossings showed a marked increase, while the swimming distance decreased significantly (*p* < 0.05, *p* < 0.01, *p* < 0.001). These results suggest that after drug administration, the rats retained a better memory of the original platform location (Figure 1A–E).

### 3.2. Effect of PAL on Neurological Function in 2VO Rats

Based on hematoxylin and eosin staining, the sham group exhibited neatly arranged neurons in the hippocampal CA1 region. These cells displayed regular pyramidal-shaped bodies, clear nuclei, evenly stained cytoplasm, and normal interstitial structure. In contrast, the model group revealed marked pathological changes in the same region. Neuronal organization appeared disordered, with some cell bodies showing swelling. The nuclei were pyknotic and deeply stained, while the cytoplasm contained vacuoles. Significant interstitial edema and widened spaces were also observed. PAL intervention effectively restored these pathological alterations. The high-dose group demonstrated more pronounced improvement, characterized by largely restored neuronal alignment, normalized cellular and nuclear morphology with typical staining, markedly alleviated interstitial edema, and essentially reconstituted tissue spaces (Figure 1F). Compared to the normal group, the model group exhibited a significant decrease in neuronal survival density within the CA1 region (*p* < 0.001). Administration of PAL markedly restored this density towards normal levels (*p* < 0.001, Figure 1G).

An extremely high neurological score was observed in the model group relative to the sham group (*p* < 0.001). The neurological score of the PAL high-dose and low-dose groups decreased markedly (*p* < 0.01, *p* < 0.05), indicating that PAL has significant neuroprotective effects (Figure 1H).

### 3.3. Effect of PAL on Plasma and Brain-Derived Metabolites in 2VO Rats

**Figure 2:**
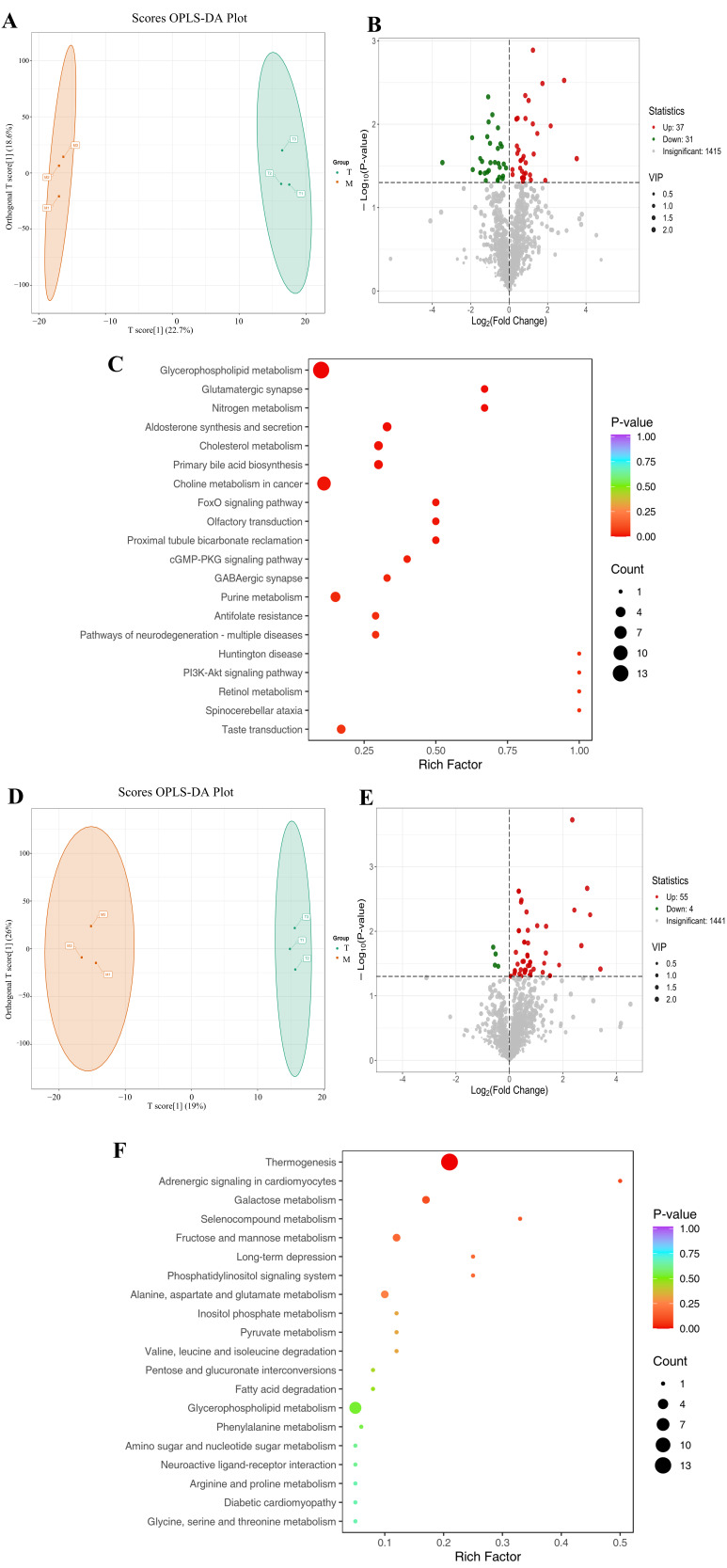
Plasma metabolomics of (**A**) OPLS-DA score plot; (**B**) Metabolite volcano plot; (**C**) KEGG pathways of differential metabolites. Brain tissue metabolomics (**D**) OPLS-DA score plot; (**E**) Metabolite volcano plot; (**F**) KEGG pathways of differential metabolites. M: Model group; T: PAL high-dose group. Animal number per group: *n* = 3.

Overall, 1483 metabolites were successfully identified in the plasma, covering substances such as amino acids, organic acids, and fatty acids. OPLS-DA was first performed on the plasma metabolomics data. In the score plot, samples from the model group and the PAL high-dose group were clearly separated (Figure 2A). A comprehensive identification and quantitative analysis of metabolites in the samples was performed. Overall, 68 potential differential metabolites were screened between the model group and the PAL high-dose group, with 37 metabolites upregulated and 31 downregulated (Figure 2B). According to the KEGG and HMDB databases, the metabolites highly associated with cognitive impairment among the differential metabolites included L-glutamate, L-glutamine, and adenosine 5′-monophosphate. The 68 differential metabolites were significantly enriched in KEGG pathways such as glycerophospholipid metabolism, glutamatergic synapse, nitrogen metabolism, aldosterone synthesis and secretion, and cholesterol metabolism (Figure 2C).

A total of 1500 metabolites were successfully identified in the brain tissue, covering substances such as amino acids, organic acids, and nucleotides. The model group and PAL high-dose group samples showed clear separation, with significant intergroup distinction (Figure 2D). Overall, 59 potential differential metabolites were screened between the model group and the PAL high-dose group, with 55 metabolites upregulated and 4 downregulated (Figure 2E). According to the KEGG and HMDB databases, metabolites highly associated with cognitive impairment among the differential metabolites included fructose-6-phosphate, mannitol, 8-hydroxyguanosine, dulcitol, and sorbitol. The 59 differential metabolites were significantly enriched in KEGG pathways such as thermogenesis, adrenergic signaling in cardiomyocytes, galactose metabolism, selenocompound metabolism, and fructose and mannose metabolism (Figure 2F).

### 3.4. Effect of PAL on Transcriptomics in 2VO Rats

**Figure 3:**
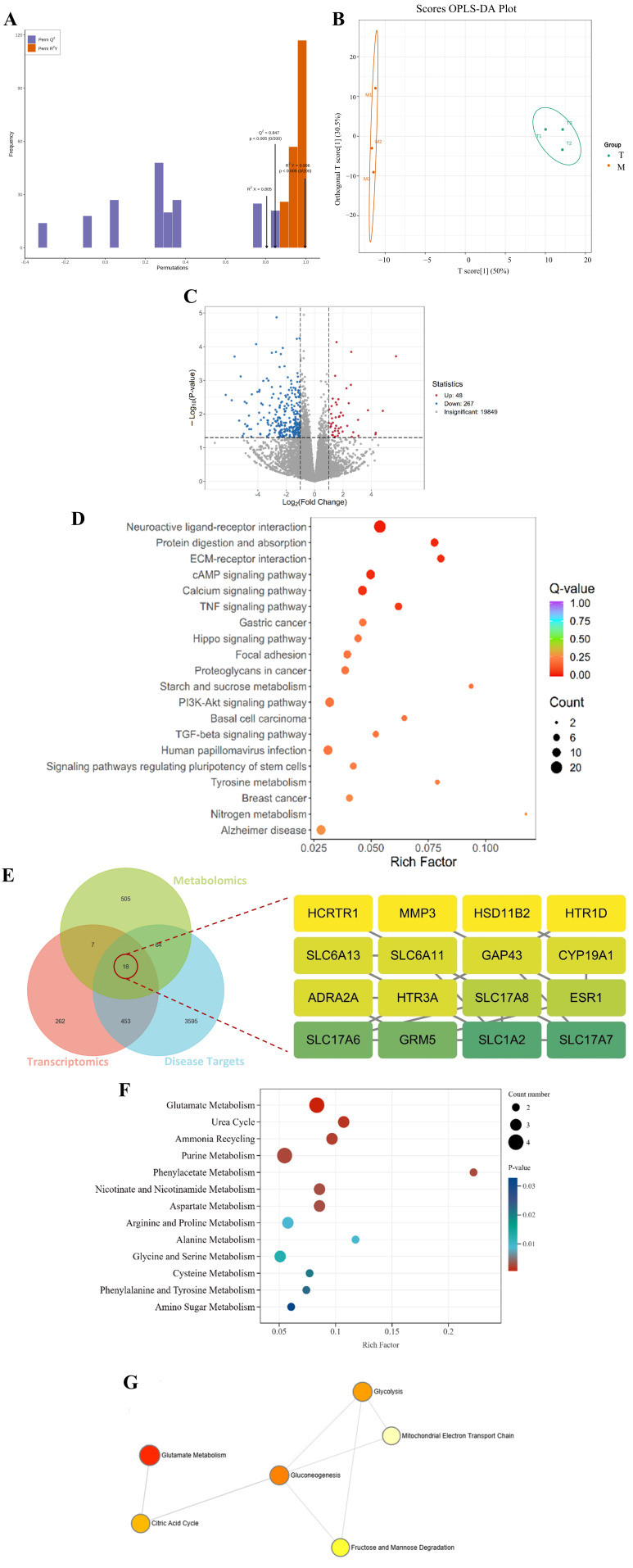
Transcriptomics of (**A**) OPLS-DA confidence interval plot; (**B**) OPLS-DA score plot (**C**) Gene volcano plot; (**D**) KEGG pathways of DEGs; (**E**) Venn diagram of jointly analyzed DEGs and PPI network; (**F**) SMPDB pathway enrichment plot of common differential metabolites; (**G**) SMPDB pathway network view of common metabolites. M: Model group; T: PAL high-dose group. Animal number per group: *n* = 3.

The Q30 base percentage for both the model group and high-dose group samples exceeded 91%, indicating a good sequencing data quality suitable for subsequent analysis. The OPLS-DA model demonstrated robust stability and explanatory power. Specifically, permutation tests indicated an excellent fit to the data, with the determination coefficient (R^2^) approaching 1, a cross-validated predictive ability (Q^2^) exceeding 0.5, and a statistically significant *p*-value < 0.05 (Figure 3A). Furthermore, the score plot revealed clear separation between the two groups (Figure 3B). The DEGs were identified by comparing the model group with the high-dose group. Comparative analysis revealed 315 genes with significantly altered expression in the high-dose group relative to the model group. Among these, 48 genes were upregulated and 267 were downregulated (Figure 3C). KEGG pathway enrichment analysis further demonstrated that the DEGs showed significant enrichment in several pathways, including neuroactive ligand–receptor interaction and protein digestion and absorption (Figure 3D).

### 3.5. Multi-Omics Integrated Analysis of PAL’s Effects on 2VO Rats

Upon combining the results of plasma and brain tissue metabolomics, a total of 710 co-existing metabolites were screened, among which 59 were significantly differential metabolites. Metabolites with an ROC AUC = 1 were selected, predicting 747 potential targets. Integration of the obtained target information with transcriptomics and disease-related targets revealed 18 common differential genes. Among these, Slc17a7 (vGLUT1) had the highest degree value in the network (Figure 3D). Further enrichment pathway association suggested that PAL might exert its therapeutic effects by regulating plasma–brain common metabolites, thereby influencing metabolic processes, such as glutamate metabolism (Figure 3E). The network view shows that glutamate metabolism is closely linked to glycolysis, the citric acid cycle, gluconeogenesis, the mitochondrial electron transport chain, as well as fructose and mannose metabolism and degradation pathways (Figure 3F).

### 3.6. Effect of PAL on Glutamate Metabolism in 2VO Rats

**Figure 4:**
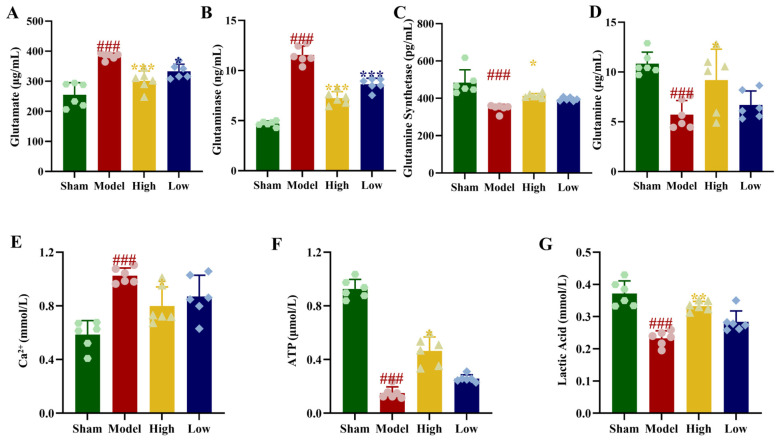
Cerebral tissue obtained from the rats in each experimental groups: (**A**) Glutamate content (Ordinary one-way ANOVA, *n* = 6); (**B**) GLS activity (Kruskal–Wallis test, *n* = 6); (**C**) GS activity (Ordinary one-way ANOVA, *n* = 6); (**D**) Glutamine content (Ordinary one-way ANOVA, *n* = 6); (**E**) Ca^2+^ content (Ordinary one-way ANOVA, *n* = 6); (**F**) ATP content (Kruskal–Wallis test, *n* = 6); (**G**) Lactate content (Kruskal–Wallis test, *n* = 6). In comparison to the sham group, ^###^ *p* < 0.001. In comparison to the model group, *** *p* < 0.001, ** *p* < 0.01, * *p* < 0.05. Data are presented as mean ± SD. High: PAL high-dose group; Low: PAL low-dose group.

Relative to the sham group, the model group exhibited higher brain levels of glutamate and Ca^2+^, along with elevated GLS activity. Concurrently, glutamine content and GS activity were lower (*p* < 0.001). Following PAL administration, the high-dose group demonstrated a significant rise in glutamine content and GS activity, coupled with a marked reduction in glutamate levels, Ca^2+^ content, and GLS activity (*p* < 0.05, *p* < 0.001). In the low-dose group, glutamate content and GLS activity were significantly decreased (*p* < 0.05, *p* < 0.001). Indicators including glutamine level, Ca^2+^ content, and GS activity demonstrated a non-significant restorative trend (Figure 4A–E).

### 3.7. Effect of PAL on Glycolysis Levels in 2VO Rats

**Figure 5:**
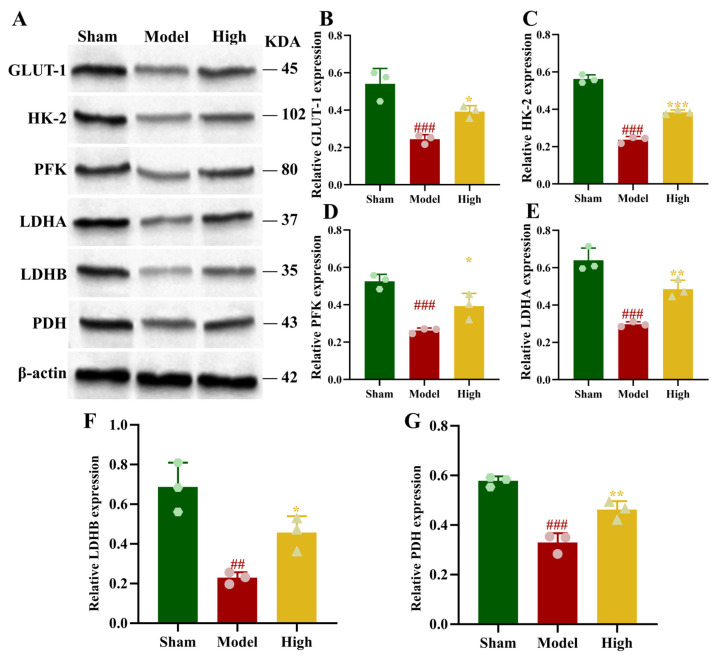
Brain tissue of rats in each group: (**A**) Blot images of glycolysis-related proteins (*n* = 3); (**B**) GLUT-1 protein expression (Ordinary one-way ANOVA, *n* = 3); (**C**) HK2 protein expression (Ordinary one-way ANOVA, *n* = 3); (**D**) PFK protein expression (Ordinary one-way ANOVA, *n* = 3); (**E**) LDHA protein expression (Ordinary one-way ANOVA, *n* = 3); (**F**) LDHB protein expression (Ordinary one-way ANOVA, *n* = 3); (**G**) PDH protein expression (Ordinary one-way ANOVA, *n* = 3). In comparison to the sham group, ^###^ *p* < 0.001, ^##^ *p* < 0.01. In comparison to the model group, *** *p* < 0.001, ** *p* < 0.01, * *p* < 0.05. Data are presented as mean ± SD. High: PAL high-dose group. The original Western blot images can be found in the Supplementary Materials.

Relative to the sham group, the model group demonstrated markedly reduced cerebral lactate and ATP content (*p* < 0.001). Following PAL administration, these levels showed significant recovery, a change particularly evident in the high-dose group (*p* < 0.05, *p* < 0.01; Figure 4F,G). The expression of glycolysis-related proteins LDHA, LDHB, GLUT-1, HK2, PFK, and PDH was notably decreased in the brain tissue of rats in the model group (*p* < 0.01, *p* < 0.001). In the PAL high-dose group, the relative protein levels of GLUT-1, HK2, PFK, LDHA, LDHB, and PDH were notably elevated compared to the model group (*p* < 0.05, *p* < 0.01, *p* < 0.001; Figure 5).

### 3.8. Effect of PAL on Glutamate Release and Lactate Shuttle in 2VO Rats

**Figure 6:**
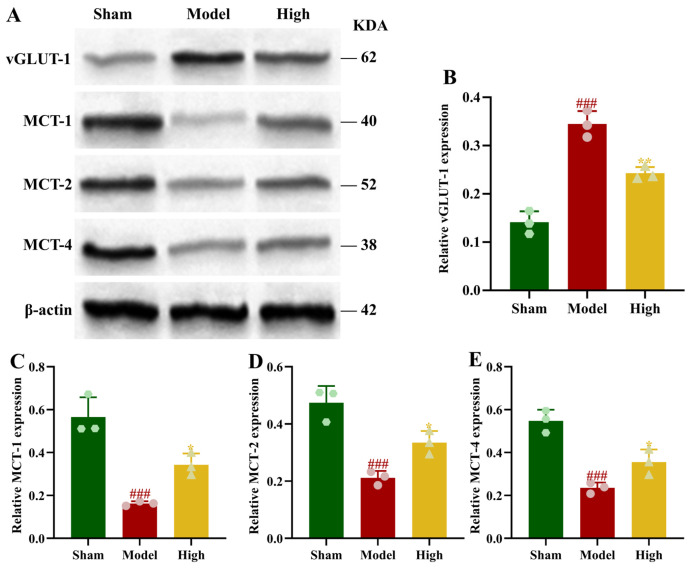
Brain tissue of rats in each group: (**A**) Blot images of proteins related to glutamate release and lactate shuttle (*n* = 3); (**B**) vGLUT1 protein expression (Ordinary one-way ANOVA, *n* = 3); (**C**) MCT1 protein expression (Ordinary one-way ANOVA, *n* = 3); (**D**) MCT2 protein expression (Ordinary one-way ANOVA, *n* = 3); (**E**) MCT4 protein expression (Ordinary one-way ANOVA, *n* = 3). In comparison to the sham group, ^###^ *p* < 0.001. In comparison to the model group, ** *p* < 0.01, * *p* < 0.05. Data are presented as mean ± SD. High: PAL high-dose group. The original Western blot images can be found in the Supplementary Materials.

A distinct pattern of change was observed in the model group relative to the sham group: the relative level of vGLUT1 protein in brain tissue was markedly increased, in contrast to the pronounced decreases seen in MCT1, MCT2, and MCT4 protein levels (*p* < 0.001). Compared to the model group, the PAL group showed a significant decrease in the relative level of vGLUT1 protein and a significant increase in the relative levels of MCT1, MCT2, and MCT4 proteins (*p* < 0.05, *p* < 0.01; Figure 6).

### 3.9. Effect of PAL on OGD-Induced Injury in Co-Cultured Cells

**Figure 7:**
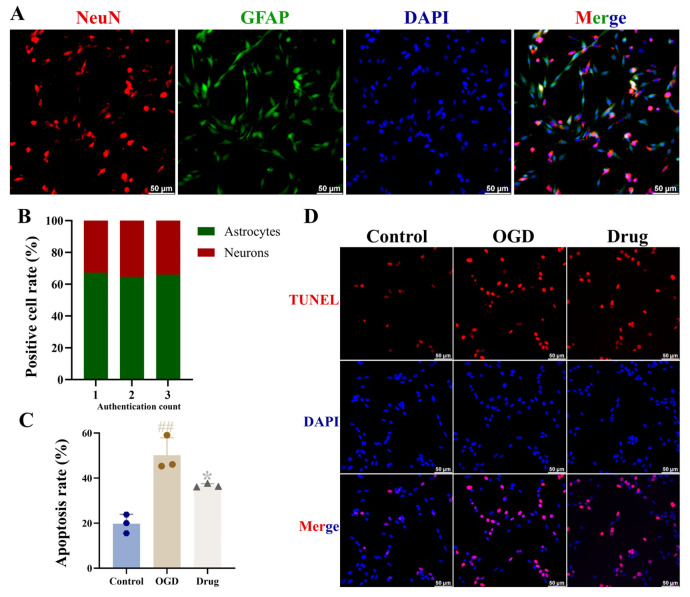
(**A**) Immunofluorescence identification images of astrocytes and neurons in co-culture (*n* = 3); (**B**) Rate of co-cultured positive cells (*n* = 3); (**C**) Apoptosis rate of co-cultured cells (Ordinary one-way ANOVA, *n* = 3); (**D**) TUNEL immunofluorescence staining images of co-cultured cells (*n* = 3). In comparison to the control group, ^##^ *p* < 0.01. In comparison to the OGD group, * *p* < 0.05. Data are presented as mean ± SD. Drug: PAL treatment group.

Identification of co-cultured cells showed that the positive rate for astrocytes and neuronal cells was 65.97 ± 1.29% and 34.03 ± 1.29%, respectively, allowing subsequent experiments to proceed (Figure 7A).

A significant increase in cell apoptosis was observed in the co-cultured astrocyte and neuronal culture system of the OGD group compared to the control group (*p* < 0.01). This was followed by a marked reduction in apoptosis in the drug (PAL) group relative to the OGD condition (*p* < 0.05; Figure 7B,C).

### 3.10. Effect of PAL on Glutamate, Lactate, and ATP Levels in the Medium of OGD Co-Cultured Cells

**Figure 8:**
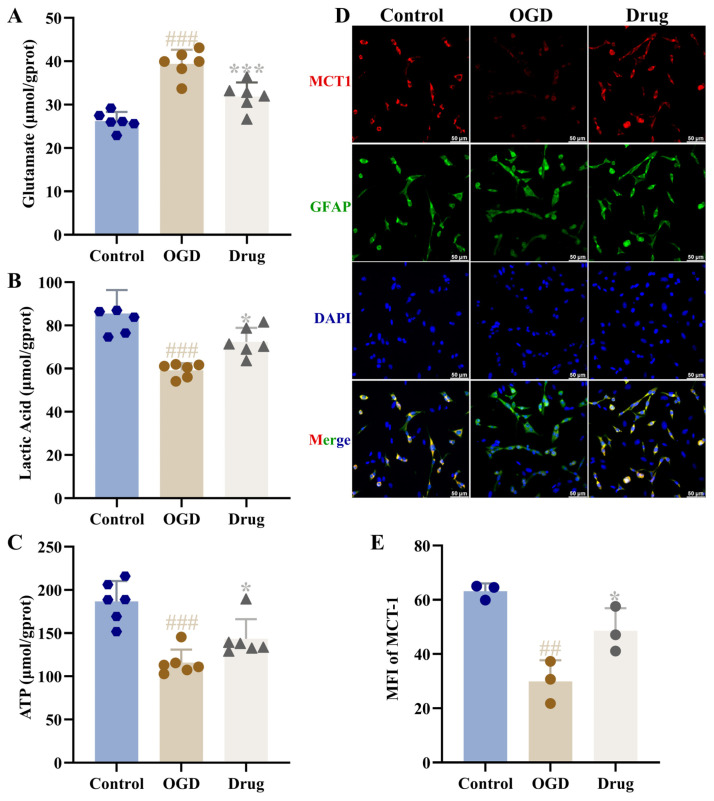
(**A**) Glutamate content in the supernatant of co-cultured cells from each group (Ordinary one-way ANOVA, *n* = 6); (**B**) Lactate content (Ordinary one-way ANOVA, *n* = 6); (**C**) ATP content (Ordinary one-way ANOVA, *n* = 6); (**D**) MCT1 immunofluorescence staining images of co-cultured cells (*n* = 3); (**E**) MCT1 immunofluorescence expression (Ordinary one-way ANOVA, *n* = 3). In comparison to the control group, ^###^ *p* < 0.001, ^##^ *p* < 0.01. In comparison to the OGD group, * *p* < 0.05, *** *p* < 0.001. Data are presented as mean ± SD. Drug: PAL treatment group.

Relative to the control group, the OGD group displayed significantly altered medium metabolite levels: glutamate content was increased, while lactate and ATP were decreased (*p* < 0.001). Following drug (PAL) administration, these changes were significantly attenuated, with glutamate content dropping and lactate and ATP levels rising relative to the OGD group (*p* < 0.05, *p* < 0.001; Figure 8A–C).

### 3.11. Effect of PAL on Glutamate Release and Lactate Shuttle in OGD Co-Cultured Cells

**Figure 9:**
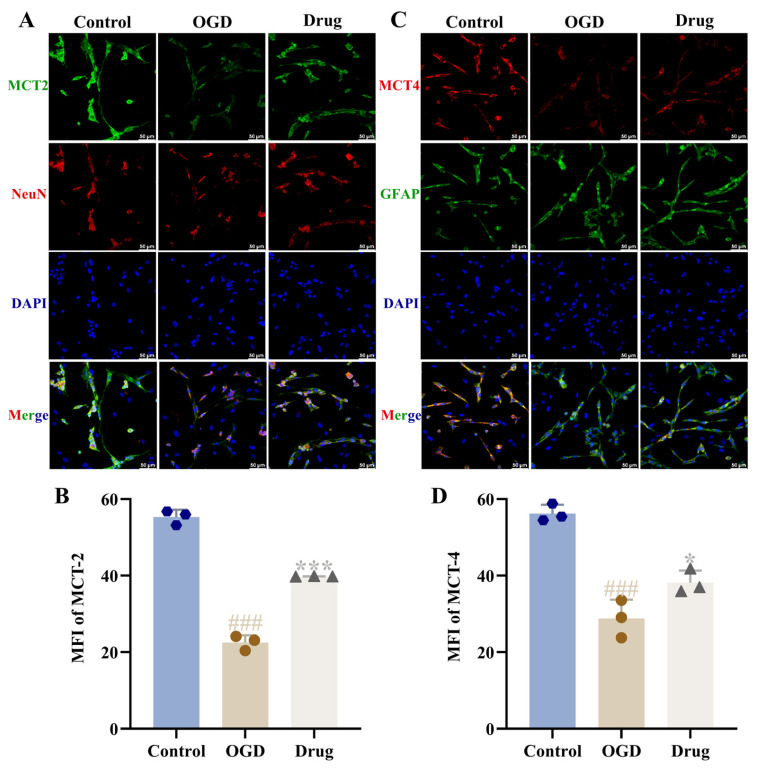
Co-cultured cells from each group: (**A**) MCT2 immunofluorescence staining images (*n* = 3); (**B**) MCT2 immunofluorescence expression (Ordinary one-way ANOVA, *n* = 3); (**C**) MCT4 immunofluorescence staining images (*n* = 3); (**D**) MCT4 immunofluorescence expression (Ordinary one-way ANOVA, *n* = 3). In comparison to the control group, ^###^ *p* < 0.001. In comparison to the OGD group, * *p* < 0.05, *** *p* < 0.001. Data are presented as mean ± SD. Drug: PAL treatment group.

In immunofluorescence staining results, we observed clear co-localization of MCT1 and MCT4 with the astrocyte marker GFAP, while MCT2 was mainly co-localized with neuronal cells. The expression distribution trends of these proteins were consistent with Western blot results. Compared to the OGD group, the drug (PAL) group showed a significant decrease in the vGLUT1 protein expression level and a significant increase in MCT1, MCT2, and MCT4 protein expression levels in the co-culture astrocyte and neuronal (*p* < 0.01, *p* < 0.001; Figure 8D,E, Figure 9). Costes’ randomization-based *p* > 0.95, indicating statistically significant colocalization.

**Figure 10:**
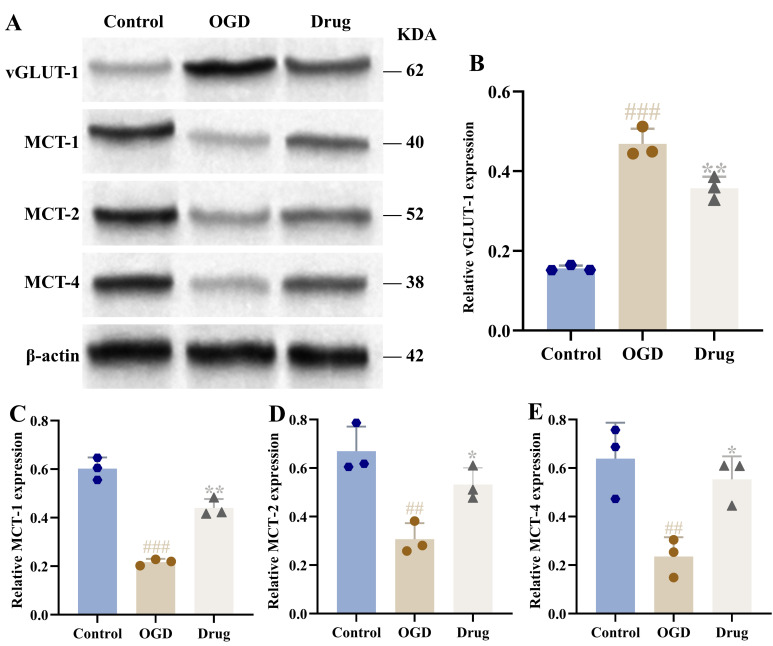
Co-cultured cells from each group: (**A**) Blot images of proteins related to glutamate release and lactate shuttle (*n* = 3); (**B**) vGLUT1 protein expression (Ordinary one-way ANOVA, *n* = 3); (**C**) MCT1 protein expression (Ordinary one-way ANOVA, *n* = 3); (**D**) MCT2 protein expression (Ordinary one-way ANOVA, *n* = 3); (**E**) MCT4 protein expression (Ordinary one-way ANOVA, *n* = 3). In comparison to the control group, ^##^ *p* < 0.01, ^###^ *p* < 0.001; In comparison to the OGD group, * *p* < 0.05, ** *p* < 0.01. Data are presented as mean ± SD. Drug: PAL treatment group. The original Western blot images can be found in the Supplementary Materials.

Relative to controls, the OGD group exhibited elevated vGLUT1 protein expression alongside reduced levels of MCT1, MCT2, and MCT4 in the co-culture astrocyte and neuronal culture system (*p* < 0.01, *p* < 0.001). This OGD-induced expression profile was attenuated by drug (PAL) intervention, which significantly decreased vGLUT1 and increased MCT1, MCT2, and MCT4 levels compared to the OGD condition (*p* < 0.05, *p* < 0.01; Figure 10).

### 3.12. Effect of PAL on Glycolysis in OGD Co-Cultured Cells

**Figure 11:**
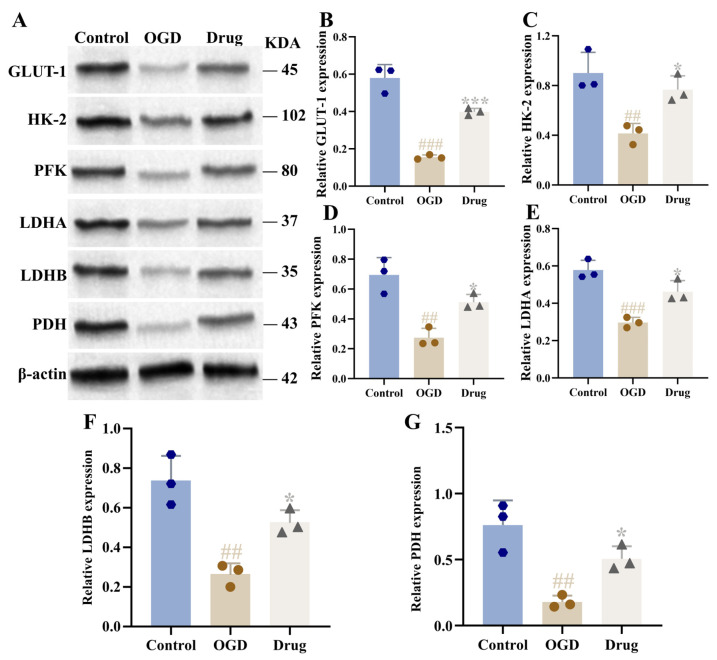
Co-cultured cells from each group: (**A**) Blot images of glycolysis-related proteins (*n* = 3); (**B**) GLUT-1 protein expression (Ordinary one-way ANOVA, *n* = 3); (**C**) HK2 protein expression (Ordinary one-way ANOVA, *n* = 3); (**D**) PFK protein expression (Ordinary one-way ANOVA, *n* = 3); (**E**) LDHA protein expression (Ordinary one-way ANOVA, *n* = 3); (**F**) LDHB protein expression (Ordinary one-way ANOVA, *n* = 3); (**G**) PDH protein expression (Ordinary one-way ANOVA, *n* = 3). In comparison to the control group, ^##^ *p* < 0.01, ^###^ *p* < 0.001; In comparison to the OGD group, * *p* < 0.05, *** *p* < 0.001. Data are presented as mean ± SD. Drug: PAL treatment group. The original Western blot images can be found in the Supplementary Materials.

The OGD group showed a significant decrease in the expression of GLUT-1, HK2, PFK, LDHA, LDHB, and PDH proteins compared to controls in the co-culture system (*p* < 0.001, *p* < 0.01). Following drug (PAL) intervention, the expression levels of these proteins were increased relative to the OGD group, restoring them towards control levels. (*p* < 0.05, *p* < 0.001; Figure 11).

## 4. Discussion

In this study, employing an integrated multi-omics strategy within a chronic cerebral hypoperfusion model, we elucidate a novel mechanism by which PAL ameliorates energy metabolism—specifically through the regulation of vGLUT1-mediated glutamate metabolism and the astrocyte–neuron lactate shuttle involving MCTs. Through the behavioral observation of rats, the pathological staining of the hippocampal CA1 region, and the detection of indicators such as TUNEL staining in vitro experiments, we found that PAL can reduce neuronal damage, reduce apoptosis and improve behavioral performance, showing that it is related to the treatment of potential cognitive dysfunction with VaD [14]. Cognitive dysfunction is one of the core symptoms of VaD, and early diagnosis and intervention are crucial to improve the prognosis of patients. By assessing cognitive damage and damage to the hippocampal neurons, the impact of drug intervention on the progression of the disease can be evaluated and provide a basis for further research [15].

In order to decipher the neural protection mechanism of PAL, we employed the shared metabolites between plasma and brain tissue as a critical link to investigate the core metabolic network through which PAL treats VaD. We have identified eight common metabolites highly related to cognitive dysfunction: L-glutamate, L-glutamine, adenosine 5′-monophosphate, fructose-6-phosphate, mannitol, 8-hydroxyguanosine, and dulcitol. Among them, L-glutamate and L-glutamine affect neurofunction and cognition by regulating neurotransmitter metabolism and energy metabolism [16,17]. Fructose-6-phosphate, mannitol and dulcitol affect the energy supply and metabolic state of cells by regulating glucose metabolism and osmotic pressure, thus affecting cognitive function [18,19,20]. 5′-monophosphate activates the AMPK/SIRT1 signaling pathway, which is anti-oxidant stress, activates autophagy and inhibits neuroinflammation, thus protecting neurons and improving cognitive function [21]. It is worth noting that 8-hydroxyguanosine, as a sign of DNA oxidative damage, reflects the level of oxidative stress. Its elevation may lead to DNA damage, damage to normal neuronal function, and aggravate cognitive dysfunction [22]. In the concentration of pathways, we found that co-metabolites are significantly enriched in glutamate metabolism, which is closely related to multiple energy metabolism pathways such as glycolysis.

Based on the above results, we speculate that PAL treatment of VaD is closely related to glutamate metabolism and energy metabolism. Further comprehensive and in-depth analysis of transcriptomics results, we found that the vesicular glutamate transporter, vGLUT1, may be one of the core molecular targets of PAL in the treatment of VaD. vGLUT1 is considered to be the key regulatory protein responsible for the loading and release of glutamate in the central nervous system. As the primary excitatory neurotransmitter, glutamate plays a key role in regulating cognitive functions, including learning and memory [23]. Under the pathological conditions of VaD, excessive glutamate often leads to neuronal damage and cognitive dysfunction. Studies show that the level of vGLUT1 is sharply upregulated in rat hippocampus with chronic hypoperfusion [24]. Overexpression of vGLUT1 will promote the excessive release of glutamic acid. This will cause glutamate to overflow the synaptic gap, trigger excitatory toxicity, eventually lead to the death of neurons, and increase the vulnerability of neurons under OGD damage [25]. Therefore, downregulating the expression of vGLUT1 can reduce the loading of glutamate into synaptic vesicles and its subsequent release, thus reducing neuronal excitability and effectively preventing nerve damage caused by excessive glutamate release.

Maintaining the dynamic balance of glutamate depends not only on the regulation of its release, but also on its timely conversion into metabolites such as glutamine [26]. Under the catalysis of GS, glutamate combines with ammonium ions to form glutamine. The increase in GS activity not only helps to remove potential neurotoxic glutamate but also produces glutamine with neuroprotective effects [27]. Under physiological conditions, glutamate and glutamine are in dynamic balance. Therefore, while enhancing GS activity, it is also necessary to inhibit the activity of GLS, reduce the conversion of glutamate into glutamine, and thus limit the production of glutamine [28,29]. Our experimental results show that PAL may reduce the release of glutamate by downregulating the activity of vGLUT1. At the same time, it promotes the decomposition of glutamate into glutamine, which ultimately reduces neuronal excitatory damage and cell apoptosis, and improves VaD.

Recently, studies have shown a tight functional coupling between glutamate metabolism and the lactate shuttle [30]. The shared metabolites from the plasma and brain tissue also suggest that PAL may exert its effects through glucose metabolism pathways. Glutamate metabolism is highly energy-consuming, requiring substantial ATP. To meet this sharp increase in energy demand, astrocytes must enhance glycolysis for rapid energy supply [31]. Energy metabolism exhaustion is also a key pathological link in cerebral chronic hypoperfusion [32].

It is crucial for astrocytes to increase lactate production to support neuronal energy supply [33]. Our in vivo and in vitro experimental results show that the protein expression in the glycolysis pathway was upregulated after PAL intervention. First, promote the glucose uptake of cells through GLUT-1 [34]. HK2 catalyzes glucose phosphorylation, which is the first step of glycolysis [35]. PFK is a key rate-limiting enzyme in glycolysis, which regulates the speed of glycolysis through the detection of ATP/AMP ratio changes [36]. Finally, LDHA is reduced to lactate, which provides energy raw materials for the shuttle. The subsequent rapid utilization of lactate is also a key link in the shuttle mechanism. The lactate ingested is oxidized back to pyruvate by LDHB [37,38]. These pyruvates are quickly sent into the tricarboxylic acid cycle by PDH, producing a large amount of ATP for neuronal cells [39]. Through the lactate shuttle mechanism, it can promote the energy supply between brain cells and effectively cope with the neuronal energy crisis in chronic hypoperfusion [40].

In addition to the production and utilization of lactate, the transmission process is equally important [41]. After astrocytes produce lactate, they should be effectively transported to neurons for use [42]. The mechanism of lactate channel refers to the transport and metabolism of lactate between astrocytes and neurons through MCTs [43]. Among various MCTs, MCT1 and MCT4 are the main targets for extracellular lactate discharge in astrocytes, maintaining intracellular lactate homeostasis and using lactate as the energy substrate of high-energy-consuming brain cells [44]. As a transporter protein with high affinity, MCT2 is expressed in neurons to promote lactate uptake [45]. After PAL intervention, the synergistic upregulation of MCT1, MCT2 and MCT4 significantly improved the transport efficiency of intracellular lactate, thus increasing the energy supply of astrocytes to neurons. We cultivated crude brain cells, among which 65.97 ± 1.29% were astrocytes, and it seems that 34.03 ± 1.29% were neurons. So, our observations reflect mainly astrocytic facet of PAL action upon hypoxia. PAL enhances the energy supply of neurons and improves the cognitive dysfunction of VaD by regulating glycolytic protein and MCT transporter and improving the lactate utilization pathway. Our current data are based on a co-culture system, reflecting cellular interactions. Future validation will require more refined cell-specific models. In addition, the absence of a positive control group in this study hampers a precise assessment of PAL’s relative efficacy. Future investigations will incorporate an active comparator to directly evaluate PAL’s efficacy profile and clinical potential.

## 5. Conclusions

By integrating data from multi-omics, we found that PAL showed significant therapeutic effects in the treatment of VaD. It can regulate vGLUT1, control the release of glutamic acid, enhance glutamic acid metabolism, and couple with glycolysis and lactate shuttle pathways. These effects jointly optimize the energy distribution between brain cells and ultimately improve cognitive function. This study clarifies the mechanism of PAL in the treatment of VaD, and provides a reliable scientific basis for the promotion of its clinical application.

## Data Availability

The transcriptomics original data presented in the study are openly available in National Center for Biotechnology Information at SUB16052610. The metabolomics original data presented in the study are openly available in China National Center for Bioinformation at OMIX015538, OMIX015539. The remaining raw data supporting the conclusions of this article will be made available by the authors on request.

## Supplementary Materials

The following supporting information can be downloaded at: https://www.mdpi.com/article/10.3390/biom16030411/s1. The original Western blot images can be found in the Supplementary Materials.
