# Supplementary material for: Investigating the Effect and Mechanism of Protocatechuic Aldehyde on Vascular Dementia Based on Multi-Omics Approach
Source: Biomolecules. 2026 Mar 11;16(3):411. doi: 10.3390/biom16030411 (PMC13024684; doi:10.3390/biom16030411)

Figure5-GLUT1

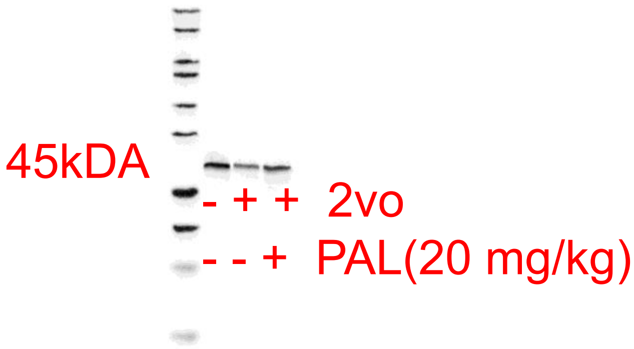

Figure5-HK2

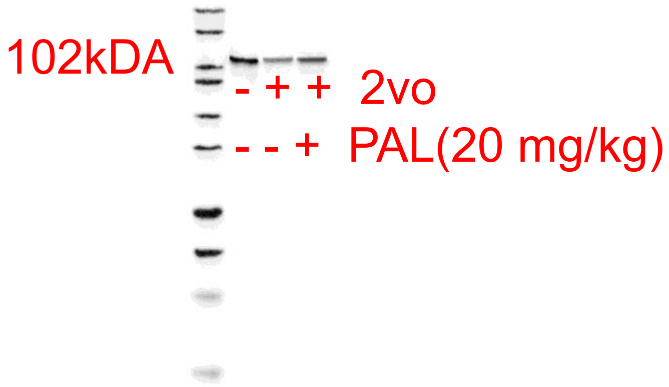

Figure5-LDHA

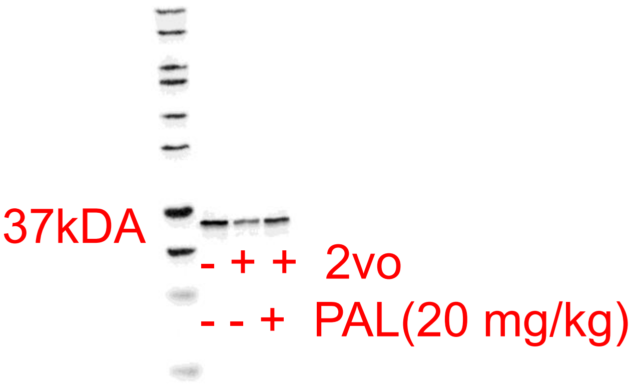

Figure5-LDHB

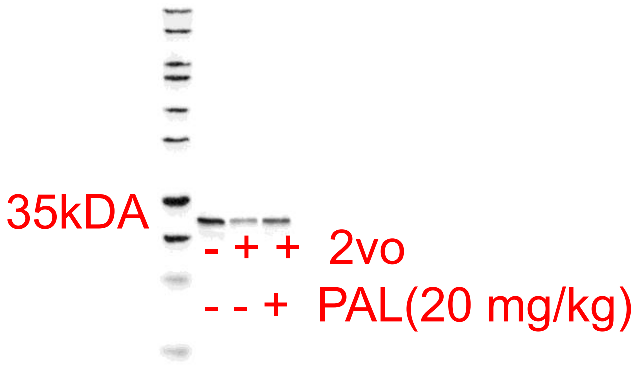

Figure5-PDH1

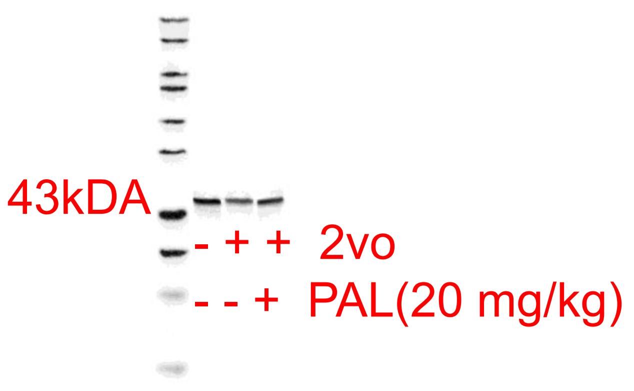

Figure5-PFK

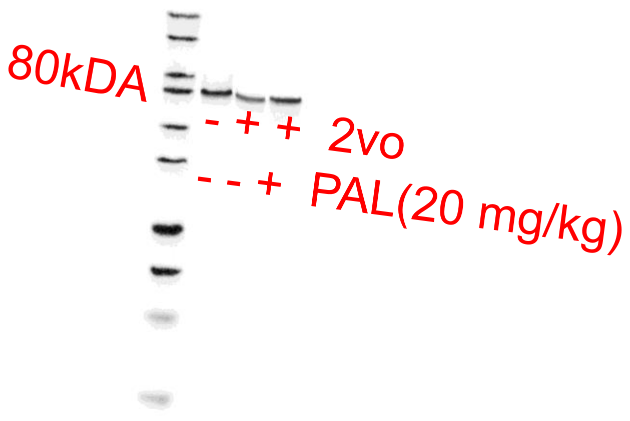

Figure5-βactin

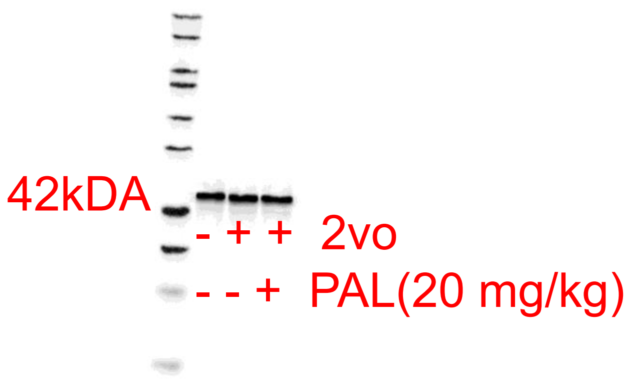

Figure6-vGLUT1

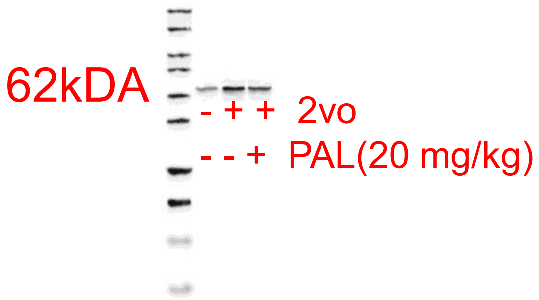

Figure6-MCT1

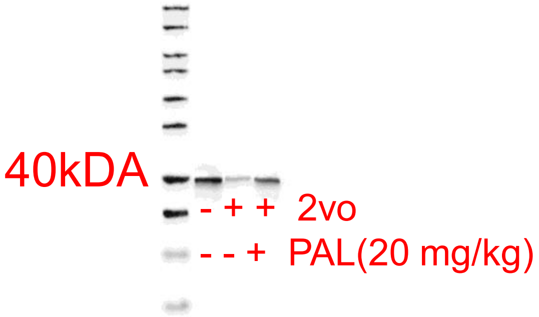

Figure6-MCT2

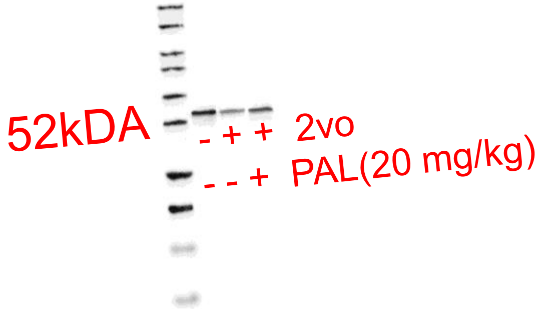

Figure6-MCT4

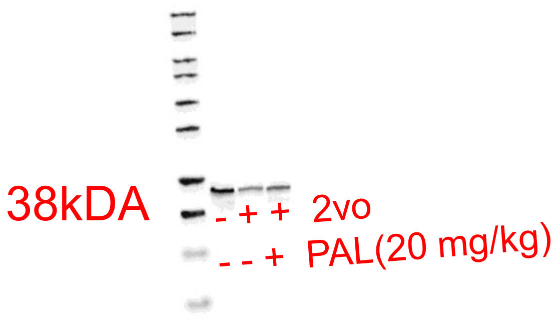

Figure6-βactin

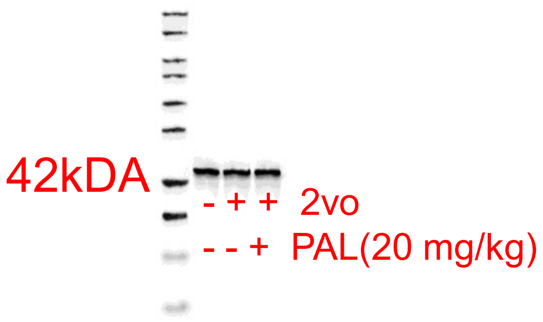

Figure10-vGLUT1

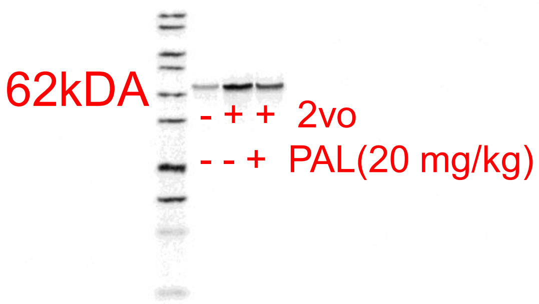

Figure10-MCT1

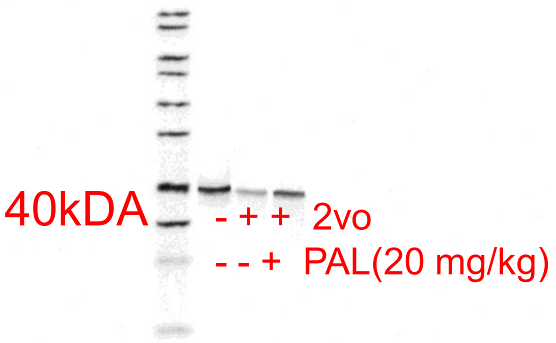

Figure10-MCT2

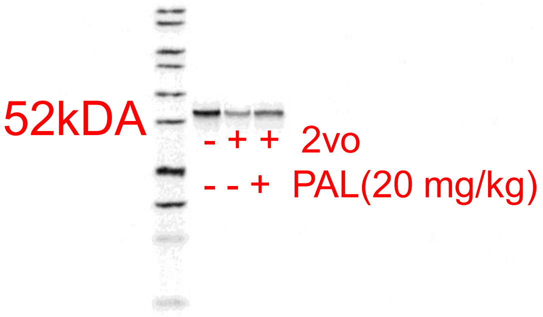

Figure10-MCT4

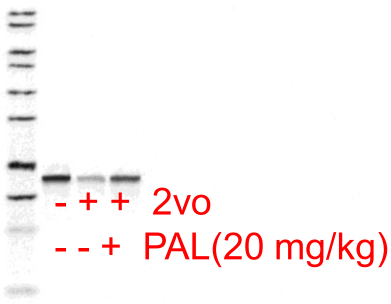

Figure10-βactin

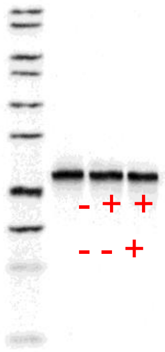

Figure11-GLUT1

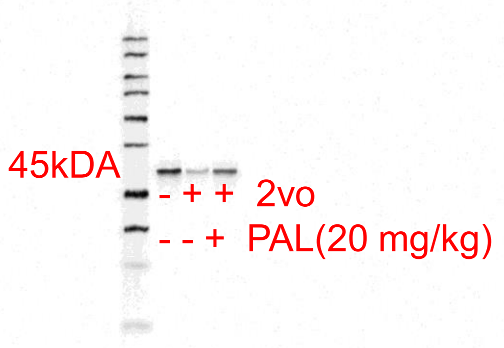

Figure11-HK2

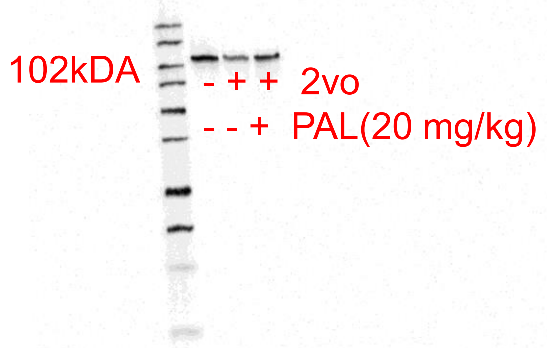

Figure11-LDHA

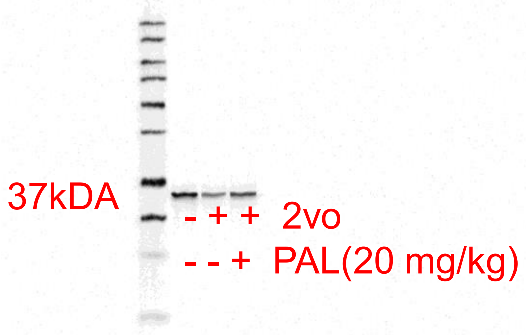

Figure11-LDHB

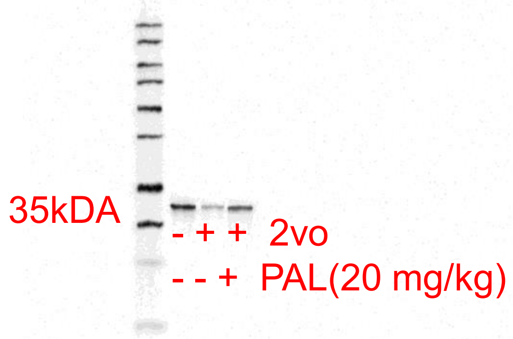

Figure11-PDH1

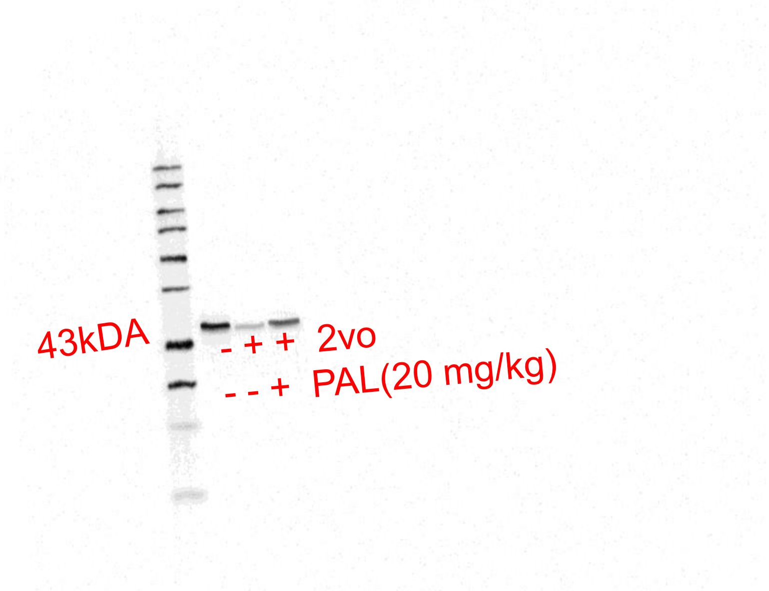

Figure11-PFK

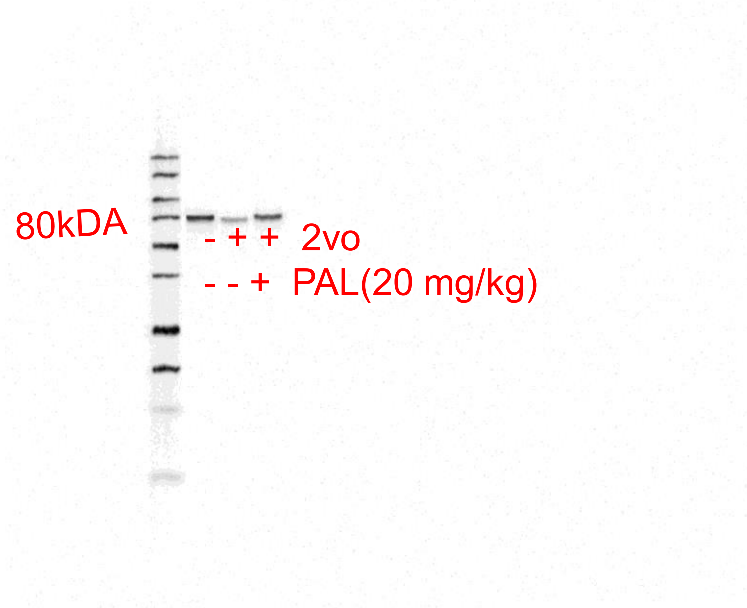

Figure11-βactin

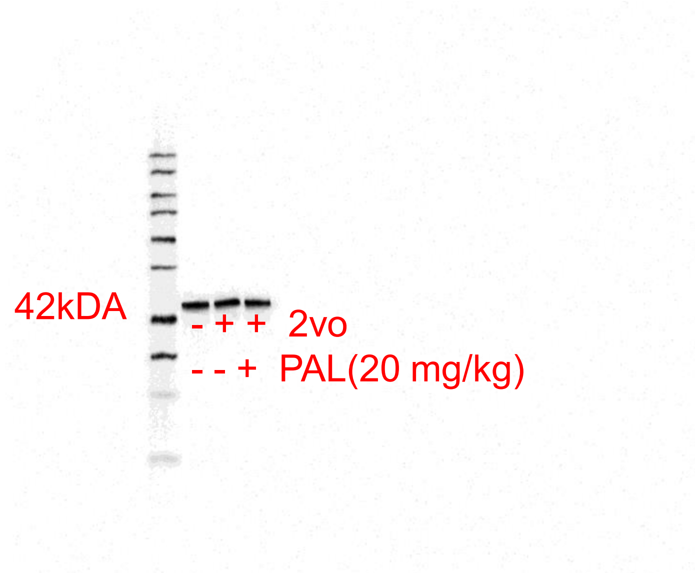

Supplement: Supplementary file 1 [file biomolecules-16-00411-s001.zip › biomolecules-4160541-supplementary.pdf]
